# Psychosocial Adjustment to Illness and Its Relationship with Spiritual Wellbeing in Iranian Cancer Patients

**DOI:** 10.1155/2020/5742569

**Published:** 2020-07-15

**Authors:** Mojtaba Senmar, Elham Hasannia, Atiyeh Moeinoddin, Shaghayegh Lotfi, Faezeh Hamedi, Mahnaz Habibi, Sajad Noorian, Hossein Rafiei

**Affiliations:** ^1^Student Research Committee, Qazvin University of Medical Sciences, Qazvin, Iran; ^2^Department of Statistics, Faculty of Science, University of Qom, Qom, Iran; ^3^Social Determinants of Health Research Center, Research Institute for Prevention of Non-Communicable Diseases, Qazvin University of Medical Sciences, Qazvin, Iran

## Abstract

**Background:**

The aim of this study was to investigate the psychosocial adjustment to illness and its relation with spiritual health in cancer patients.

**Methods:**

This study was conducted in 2019 in Iran. It was a descriptive study with a sample of 124 cancer patients. Data were collected using two questionnaires of the psychosocial adjustment to illness scale (PAIS) with 46 questions and the Paloutzian and Ellison spiritual health scale with 20 questions.

**Results:**

The mean age of the participants in this study was 52.4 ± 13.2 (range 18 to 87 years). The mean months of life with cancer were 16.5 months. The mean score of psychosocial adjustment to illness was 30.7 ± 15.5. The mean score of spiritual wellbeing in the studied patients was 71.4 ± 17.1. The results of the Pearson correlation test showed a significant inverse relationship between the mean score of psychosocial adjustment to illness and the mean score of spiritual wellbeing (*p* > 0.001, rr = −.355).

**Conclusion:**

Cancer patients in this study had relatively good psychosocial adjustment with their illness. Spiritual wellbeing can increase psychosocial adjustment to illness in this group of patients.

## 1. Introduction

Studies estimate that there are about 18.1 million new cancer cases worldwide in 2018 that caused 9.6 million deaths this year [1]. Cancer usually needs severe and prolonged treatment and can encounter patients with mental and physical crises [2]. Therefore, in patients with cancer, psychosocial adjustment to illness is important [3]. Cancer adaptation continues from the cancer diagnosis to the end of treatment [4]. Inappropriate psychosocial adjustment to illness in cancer patients can lead to several complications such as poorer health outcomes, reduced adherence to treatment, and disease-related care [5].

Given the importance of the subject, previous studies were interested in examining psychosocial adjustment to illness among cancer patients. In a study in 2017, researchers examined the psychosocial adjustment to illness among breast cancer patients. The results of that study showed that women with breast cancer had a low level of psychosocial adjustment to illness [3]. In another study in this regard, Taghadosi et al. examined the psychosocial adjustment to illness among 260 cancer patients. Results of the Taghadosi et al. study showed that most patients had a moderate to high level of adaptation to their disease [5]. In another study in 2016 in Nepal, researchers examined the psychosocial adjustment to illness among patients with ostomy using the Ostomy Adjustment Inventory-23. The results of this study showed that this group of patients had a relatively moderate level of psychosocial adjustment to illness [6].

Spiritual care as part of holistic care, in addition to paying attention to the patient's religious beliefs and practices, encompasses other concepts such as belief and faith in self, others and, for some, a deity or higher power creativity and self-expression, forgiveness, hope and strength, love and relationships, meaning and purpose, morality, peoples' values, and trust that should be considered by nurses when providing spiritual care [7, 8]. In recent years, more attention has been paid to the role of spirituality as a mechanism of coping with disease in patients with chronic diseases. In a study conducted by Sajadian et al. among cancer patients, patients reported that seeking social support, spirituality, and positive cognitive restructuring and making changes are the most coping strategies used by them for better adjustment to illness [4]. Patients in the Sajadian et al. study also reported that over time, they used more spirituality and problem solving strategies than other strategies for adjustment to illness [4]. In another study, Moghaddam et al. reported that one of the ways to increase adjustment to illness in cancer patients is to utilize and enhance one's spiritual wellbeing [3].

Although limited previous studies revealed the role of spirituality in adjustment to illness in cancer patients, this issue has not been thoroughly explored in cancer patients in Iran. Difference in religious belief (most people in Iran are Muslims) and cultural and social support of cancer patients in Iran compared to other countries may affect patients' psychosocial adjustment to illness and its related factors. The present study was conducted to examine the psychosocial adjustment to illness and its relationship with spiritual health in Iranian cancer patients.

## 2. Methods

The present cross-sectional study was performed in the oncology ward of the Qazvin Velayat Hospital. The Velayat Hospital is a public hospital affiliated to the Qazvin University of Medical Sciences, where most patients with cancer are treated in the province. Patients with different types of cancer are admitted and treated in this ward.

By using a sample size formula, we needed at least 97 patients. Because of the availability of our patients, we selected 124 patients by using a convenience sampling method. Questionnaires were distributed by three of the researchers. For this purpose, the researchers visited the oncology ward daily in the morning shift and after coordination with the head nurse invited all patients with inclusion criteria to participate in the study. This took about 3 months. Questionnaires were provided to patients at rest time. Before completing the questionnaires, the researchers provided the patients with the necessary explanations about the study objectives and the questionnaires and how to complete them. They were then asked to complete the questionnaires and then put them in a package provided to them which was returned to the researcher. When the questionnaires were completed by patients, researchers were present in the oncology ward to answer participants' questions.

## 3. The Tools Used in This Study

### 3.1. Demographic Checklist

This researcher-made checklist includes items such as age, gender, marital status, type of cancer, type of treatment, having other illnesses, economical status, and level of education.

### 3.2. The Paloutzian and Ellison Spiritual Wellbeing Scale

To assess the spiritual wellbeing of the patients in the present study, the 20-item Paloutzian and Ellison scale was used [9]. This scale has two main dimensions (religious wellbeing and existential wellbeing) that have 10 questions each. All 20 questions are answered in a six Likert-type scale from completely agree to completely disagree. Some questions in this questionnaire are reversed. The final score of this questionnaire, which is the sum of the two-dimensional score, is between 20 and 120. A higher score on this questionnaire indicates higher spiritual wellbeing. This questionnaire has been well translated and validated in previous studies in Iran [10].

### 3.3. Psychosocial Adjustment to Illness Scale (PAIS)

PAIS was developed by Derogatis and has 46 questions that are divided into 7 subscale includes: health care orientation, vocational environment, domestic environment, sexual relationship, extended family relationship, social environment, and psychological distress [11]. Questions are answered in a four-point Likert scale. The score of each subscale was summed to determine the total score. A higher score in this questionnaire indicates lower psychosocial adjustment to illness. This questionnaire has been well translated and validated in previous studies in Iran [12].

### 3.4. Data Analysis

Data analysis was performed by a Statistical Specialist (SN) member of the research team. Data were entered into SPSS 16 software. The statistical tests used were Pearson's correlation test (to determine the relationship between two quantitative variables), independent sample *t*-test (to determine mean differences between two categorical groups), and one-way ANOVA (to determine mean differences between three or more than three categorical groups). *p* value less than 0.05 was considered significant.

## 4. Results

The mean age of the participants was 52.4 ± 13.2 (range 18 to 87 years). 92 participants were female and the rest were male. Most types of cancer were breast cancer (46 patients). The mean months of cancer were 16.5 months.  Table 1 shows the demographic information in more detail.

### 4.1. Psychosocial Adjustment to Illness

The mean score of psychosocial adjustment to illness was 30.7 ± 15.5. The mean scores of health care orientation, vocational environment, domestic environment, sexual relationship, extended family relationship, social environment, and psychological distress subscales were 27.5, 43.8, 33.3, 35.8, 16.6, 29.2, and 35.9, respectively. The mean score of psychosocial adjustment to illness was 29.9 in women and 33.2 in men (*p* = 0.30). There was no significant relationship between patients' age and psychosocial adjustment to illness (*p* = 0.057). With increasing patient education level, the mean score of psychosocial adjustment to illness was significantly decreased (*p* = 0.015). There was no significant difference between the mean scores of psychosocial adjustment to illness for single and married patients (*p* = 0.767). The mean score of psychosocial adjustment to illness was significantly lower in those with better economic status (*p* = 0.004). The mean score of psychosocial adjustment to illness was not significantly different between individuals with different stages of the disease (*p* = 0.482). The results of the Pearson correlation test showed no significant relationship between the mean score of psychosocial adjustment with illness and duration of disease (*p* = 0.075).

### 4.2. Spiritual Wellbeing

The mean scores of total wellbeing, religious wellbeing, and existential wellbeing were 71.4, 80.2, and 62.7, respectively. The mean score of spiritual wellbeing was 72.21 in females and 69.22 in males (*p* = 0.274). There was no significant relationship between patients' age and their spiritual wellbeing (*p* = 0.058). Patients with a higher education level had higher spiritual wellbeing significantly (*p* = 0.029). There was no significant difference between the mean scores of spiritual wellbeing in single and married women (*p* = 0.450). There was no significant difference in mean score of spiritual wellbeing among patients with different stages of the disease (*p* = 0.721). The results of the Pearson correlation test did not show significant relationship between mean scores of spiritual wellbeing and duration of disease (*p* = 0.090).

### 4.3. Relationship between Psychosocial Adjustment to Illness and Spiritual Wellbeing

**Figure 1:**
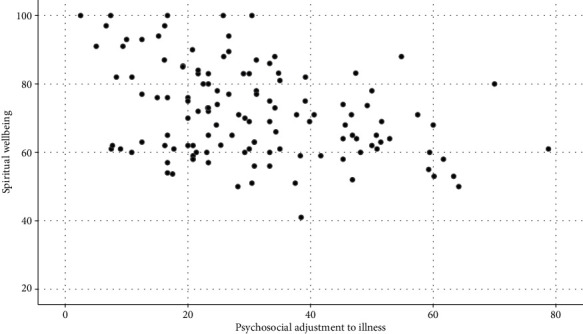


Results of the Pearson correlation test showed an inverse and significant relationship between the mean score of psychosocial adjustment to illness and the mean score of spiritual wellbeing (*p* < 0.001, rr = −.355).  Figure 1 shows the distribution of these two variables. In Table 2, the relationship between psychosocial adjustment to illness and spiritual wellbeing is shown in more detail.

## 5. Discussion

Psychosocial adjustment to illness is vital in many chronic diseases, including cancer. The results of the present study showed that cancer patients have relatively good psychosocial adjustment to their illness. Psychosocial adjustment to illness in the present study was related to the patient's spiritual wellbeing, education level, and economic status significantly.

Literature review showed three studies that examined the relationship between psychosocial adjustment with illness and spiritual wellbeing in cancer patients, two studies on patients with intestinal ostomy and one study on patients with leukemia. In the first study, Li et al., in Taiwan, examined these two concepts among 45 patients with intestinal stoma. The tools used in this study were the same as those used in the present study to assess psychosocial adjustment to illness and spiritual health. Similar to the results of the present study, the results of the Li et al. study showed that psychosocial adjustment to illness had a significant relationship with the patient's level of spiritual wellbeing [13]. The results of our study are different in some aspect compared to the Li et al. study. In their study, Li et al. reported that from two dimensions of spiritual wellbeing (religious and existential), only existential wellbeing was significantly associated with psychosocial adjustment to illness and its dimensions. However, religious wellbeing has no relationship with psychosocial adjustment to illness and its dimensions. In the first part, results of our study also showed significant relationship between existential wellbeing and psychosocial adjustment to illness and its dimensions that is similar with results of Li et al.; however, in the second part, our study showed that religious wellbeing has significant relationship with some dimensions of psychosocial adjustment to illness which includes health care orientation, vocational environment, and domestic environment. The difference between the results of the two studies could be related to differences in religious beliefs in the two groups. All participants in our study were Muslims, and there are specific religious beliefs that influence their religious health [2]. In this regard, Howsepian and Merluzzi reported that cancer patients' religious beliefs may not directly affect their psychosocial adjustment to illness; however, patients who have a specific religious belief usually received more social support from their religious groups that increase their psychosocial adjustment to illness [14]. In the second study conducted in 2019, in Turkey, Ayik et al. examined the relationship between spiritual wellbeing and psychosocial adjustment to illness among 95 ostomy patients. The study used two questionnaires of Functional Assessment of Chronic Illness Therapy-Spiritual Well-Being Scale and the 23-item Ostomy Adjustment Scale to assess spiritual wellbeing and psychosocial adjustment to illness. Similar to the results of the present study, the Ayik et al. study showed that patients with higher spiritual wellbeing had better psychosocial adjustment to illness [15]. In the third study, O'Connor et al. examined the relationships between spiritual wellbeing and psychological adjustment to illness among 40 patients with leukemia. O'Connor et al. used a different questionnaire and reported that spiritual wellbeing enhanced psychological adjustment to illness in this group of patients [16].

The results of the present study also showed that people with better economic status as well as those with higher education have more adjustment to illness. Previous studies also showed a similar finding in this regard. Li et al. report that patients who experienced more economic problems during cancer treatment have a lower level of psychosocial adjustment to illness [13]. Cancer is a costly disease that, in some cases, especially in developing countries where weaker financial support systems exist for patients, affects the treatment and all aspects of the patient's life. Taghadosi et al. reported that patients with a higher education level had a higher level of psychosocial adjustment to illness compared to patients with a lower level of education [5]. There are a number of reasons for the relationship between the patient's education level and psychosocial adjustment to illness. For example, the results of a study showed that cancer patients with a higher level of education had a higher level of self-efficacy compared to those with lower education. Higher self-efficacy also made people adjust more to their illness [17].

## 6. Conclusion

The results of this study showed that cancer patients had a relatively good level of psychosocial adjustment to illness. Spiritual health can improve psychosocial adjustment to illness in cancer patients. Health care providers should consider this in time of caring for patients and provide the basis for improving the living conditions of this group of patients. Due to the lack of similar studies, it is strongly recommended to conduct studies in this area.

## Figures and Tables

**Table 1:** Patient demographic characteristics.

Gender	Men	34 (27%)
	Women	92 (73%)

Age		52.4 ± 13.7

Marital status	Single	8 (6.3%)
	Married	106 (84.1%)
	Widowed and divorced	12 (9.5%)

Education level	Illiterate	28 (22.2%)
	Under diploma	61 (48.4%)
	Diploma	26 (20.6%)
	Higher diploma	11 (8.7%)

Economic status	High	9 (7.1%)
	Moderate	81 (64.3%)
	Low	36 (28.6%)

Type of treatment	Surgery	60 (47.6%)
	Chemotherapy	121 (96%)
	Radiotherapy	23 (18.3%)

Cancer stage	Stage 1	26
	Stage 2	38
	Stage 3	14
	Stage 4	11

**Table 2:** Relationship between psychosocial adjustment to illness and spiritual wellbeing.

	Existential wellbeing	Religious wellbeing
Health care orientation	rr = −.282; *p* = 0.001	rr = −.254; *p* = 0.004
Vocational environment	rr = −.283; *p* = 0.04	rr = −.337; *p* = 0.024
Domestic environment	rr = −.501; *p* < 0.001	rr = −.174; *p* = 0.04
Sexual relationship	rr = −.235; *p* = 0.014	rr = .113; *p* = 0.246
Extended family relationship	rr = −.207; *p* < 0.015	rr = −.030; *p* = 0.737
Social environment	rr = −.323; *p* < 0.001	rr = −.008; *p* = 0.268
Psychological distress	rr = −.504; *p* < 0.001	rr = −.012; *p* = 0.896
Psychosocial adjustment total score	rr = −.503; *p* < 0.001	rr = −.099; *p* = 0.268

## Data Availability

The data used to support the findings of this study are available from the corresponding author upon request.

## Abbreviations

PAIS: Psychosocial adjustment to illness scale
ANOVA: Analysis of variance.
